# Adipokines and Endothelium Dysfunction Markers in Pregnant Women with Gestational Hypertension

**DOI:** 10.1155/2019/7541846

**Published:** 2019-10-13

**Authors:** Elżbieta Poniedziałek–Czajkowska, Radzisław Mierzyński, Dominik Dłuski, Bożena Leszczyńska-Gorzelak

**Affiliations:** Department of Obstetrics and Perinatology, Medical University of Lublin, ul. Jaczewskiego 8, 20-094 Lublin, Poland

## Abstract

**Objective:**

The aim of the study was to evaluate the levels of adipokines such as adiponectin and leptin as well as soluble intercellular adhesion molecule-1 (sICAM-1) and endogenous NOS inhibitor-asymmetric dimethylarginine (ADMA), as the endothelium dysfunction markers in pregnant women with gestational hypertension (GH).

**Patients and Methods:**

Adiponectin, leptin, sICAM-1, and ADMA concentrations were measured in a group of 34 patients with GH and in 32 healthy pregnant women between the 24^th^ and 34^th^ week of gestation with ELISA tests.

**Results:**

The patients with GH compared with healthy ones were characterized by significantly higher BMI (28.09 ± 7.90 vs. 22.34 ± 4.21 kg/m^2^, *p*=0.016) and higher concentrations of leptin (45.89 ± 35.91 vs. 24.09 ± 24.40 ng/mL, *p*=0.006). sICAM-1 levels were also higher in the GH group but without the statistical significance (264.51 ± 50.99 vs. 232.56 ± 43.3 ng/ml, *p*=0.057). There were no significant differences between groups in adiponectin (8.79 ± 8.67 vs. 7.90 ± 3.71 *μ*g/mL, *p*=0.46, NS) and ADMA (0.57 ± 0.26 vs. 0.60 ± 0.24 *μ*mol/L, *p*=0.68, NS) levels. The significant correlation between leptin levels and BMI value was observed only in patients with GH (*R* = 0.56, *p*=0.02).

**Conclusions:**

The higher levels of leptin in pregnant women with gestational hypertension may be suggestive of the role of leptin in GH development. As the patients in the GH group had higher BMI, hyperleptinemia may link obesity with gestational hypertension. The significance of leptin as the predictive marker of GH development could be implied. It could be postulated that the higher levels of sICAM-1 in the GH patients, although not statistically significant, could reflect some impairment of the endothelium function occurring in GH regardless of BMI. The comparable adiponectin levels in GH and healthy pregnant patients and the lack of its correlation with BMI may indicate the occurrence of a protective mechanism in pregnancy maintaining its concentration and preserving from the consequences of the decrease in its levels in overweight and obese patients. Since ADMA levels were similar in GH and healthy pregnant women, ADMA seems not to be involved in GH pathogenesis, suggesting that NO synthesis is not impaired in this pregnancy complication. As the data on the gestational hypertension pathogenesis and its correlations with adipokines and markers of the endothelium dysfunction are limited, further studies on this issue are warranted.

## 1. Introduction

The American College of Gynecologists and Obstetricians presented the classification of hypertensive disorders during pregnancy which is widely accepted and includes gestational hypertension (GH), preeclampsia (PE)-eclampsia, chronic hypertension, and preeclampsia superimposed on chronic hypertension [1]. Hypertensive disorders of pregnancy affect up to 10% of pregnancies worldwide: approximately 3–9% of all pregnant women suffer from GH and 2-3% from preeclampsia [2]. Hypertensive disorders during pregnancy are not only the major cause of maternal and neonatal mortality and morbidity but also constitute risk factors for women and their offspring for cardiovascular disease development later in life [3–5].

There are several recognized risk factors for GH and PE, including chronic diseases such as obesity, hypertension, and diabetes mellitus [6]. Epidemiological studies have revealed that obesity (defined as a body mass index of ≥ 30 kg/m^2^) is linked with an up to fivefold increase in the rate of preeclampsia [7].

There are a lot of theories attempting to elucidate the reasons and the pathogenesis of the hypertension development during pregnancy, mainly preeclampsia. It has been proposed that the final pathophysiological path leading to preeclampsia is the deficient trophoblastic invasion and failure of the remodeling of uterine spiral arteries which results in placental ischemia and the consecutive release of factors inducing the activation and dysfunction of maternal endothelium. Finally, it leads to the impairment of the balance between vasoconstrictor and vasodilator agents and to the maternal systematic inflammatory response [8–10].

The involvement of adipokines, such as adiponectin and leptin, and the role of oxidative stress in PE development are also postulated [11–13].

Adipocytokines (adipokines) are the family of various proteins synthesized and secreted by adipocytes. These proteins are engaged in the control and regulation of different processes like the appetite, energy balance, insulin sensitivity, glucose and lipid metabolism angiogenesis, inflammation, and blood pressure. Their synthesis is altered in obesity, type 2 diabetes, and metabolic syndrome [14]. Leptin and adiponectin, which represent the adipocytokines group, are known to be synthesized not only by adipose tissue but also within the intrauterine environment [15].

Leptin, the product of the *ob* gene is a 16-kDa protein hormone which is involved in the regulation of energy balance and, apart from pregnancy, is almost exclusively synthesized by the white adipose tissue [15]. The concentrations and adipose tissue mRNA expression of leptin are strongly associated with BMI and the fat mass [16]. The functions attributed to leptin are extensive: it is involved in the regulation of the endocrine system, inflammation processes, immune response, reproduction, and angiogenesis. Leptin is postulated to play an important role in the implantation through the modulation of trophoblastic growth factors and by inducing invasive metalloproteinases [17]. Belonging to the type I cytokine superfamily and possessing the structure similar to interleukin-6, leptin can also be considered as a proinflammatory cytokine [18].

During pregnancy, the leptin levels are two- to three-folds higher as compared with nonpregnancy conditions with the peak observed about the 28^th^ week and they decrease to the pregravid concentrations immediately after delivery. The placenta is a significant source of the maternal circulating leptin, which is positively correlated with the fat mass during pregnancy [19]. Although the leptin levels typically correlate with adipose tissue mass, it has been shown that obese individuals had the elevated leptin levels without expected anorexic responses. This observation could suggest the leptin resistance in obesity [20]. Pregnancy, predominantly in the overweight and obese pregnant women, is considered as a leptin-resistant state as well [21].

There is a growing body of evidence that leptin may play a direct role in the pathogenesis of preeclampsia. The increased leptin levels lead to hypertension in mouse models and have been shown to increase blood pressure through sympathetic activation and by influencing nitric oxide synthesis [22, 23]. It has also been demonstrated in an animal model (female mice) that leptin induced hypertension and endothelial dysfunction via aldosterone-dependent mechanism [24].

Furthermore, leptin is believed to have proinflammatory properties, and inflammation is associated with preeclampsia [9, 25]. The higher leptin levels in PE could result from placental stress to increase nutrient delivery to the fetus or it could be related to an augmented leptin expression by the hypoxic placenta in preeclampsia [26, 27].

Adiponectin, a 30-kDa protein synthesized almost exclusively by adipocytes, is thought to be an insulin-sensitizing, anti-inflammatory, and antiatherogenic adipokine [28, 29] with antioxidant and angiogenic properties [28–30]. The adiponectin expression is inhibited by proinflammatory cytokines, hypoxia, and oxidative stress [31].

The adiponectin levels are decreased in obesity, type 2 diabetes mellitus, insulin resistance, and hypertension [32]. It has been observed that in pregnancy, the maternal adiponectin secretion progressively declines. Both the plasma adiponectin concentrations and mRNA expression are negatively correlated with the fat mass what is suggestive that adipose tissue accretion is associated with signals for the restriction of adiponectin production [33]. The results of the studies on the adiponectin concentrations in PE are inconclusive; some of them have revealed the significant increase in circulating adiponectin levels but some of them have not [34–37].

The endothelial cell dysfunction has been considered crucial to PE pathophysiology, since it may trigger hemostatic and inflammatory systems and results in abnormal placentation [38]. The abnormal placentation which is considered as a starting point for the PE development leads to placental ischemia and hypoxia: it results in the release of factors responsible for the endothelial dysfunction. The reports have shown the imbalance between the proangiogenic factors like vascular endothelial growth factor (VEGF) and placental growth factor (PlGF) and the antiangiogenic factors such as soluble fms-like tyrosine kinase-1 (sFlt-1) and soluble endoglin (sEng). Proinflammatory cytokines such as tumor necrosis factor-*α* (TNF-*α*) and interleukin-6 (IL-6) and hypoxia-inducible factor (HIF), reactive oxygen species and angiotensin II type 1 receptor, all are involved in the development of the endothelial dysfunction and preeclampsia [39–41].

The term “endothelial dysfunction” has also been used in the context of the disturbed endothelium-dependent vasodilatation caused by loss of nitric oxide (NO) in which bioactivity could be considered as the key factor for its development [42]. Nitric oxide is responsible not only for the vasodilation but it is also involved in various favorable processes within the vessel wall, such as the reduction of the vascular smooth muscle tone, cell migration and growth, platelet aggregation and thrombosis, monocyte and macrophage adhesion, and inflammation [43]. It is the main vasodilator in the placenta, affecting the vascular reactivity of fetoplacental unit, trophoblast invasion and apoptosis, and platelet adhesion and aggregation in the intervillous space [44].

Asymmetric dimethylarginine (ADMA), which is a posttranslationally modified form of arginine, is a competitive inhibitor of L-arginine for all NO synthase (NOS) isoforms. ADMA contributes to the endothelial dysfunction and diminishes endothelium-related vasodilatation, and it is recognized as a biomarker of endothelial and cardiovascular disorders [45, 46]. Maternal plasma levels of ADMA are decreased in physiological pregnancy but increase as the gestational age increases. Its higher levels have been found in preeclampsia [47]. The augmented ADMA concentrations at the beginning of pregnancy correlate with the endothelial dysfunction and could be predictive for PE development [48].

The endothelial condition could be assessed with the use of endothelial activation markers, such as soluble vascular cell adhesion molecule (sVCAM), soluble intercellular adhesion molecule (sICAM), endothelin 1 (ET-1), E-selectin, and others. Adhesion molecules play a central role in the endothelial cells-leukocytes adherence and the subsequent migration of white blood cells to perivascular tissue. An increase in sVCAM-1 and sICAM-1 levels indicates the endothelial cell activation/dysfunction and has been implicated in the PE pathophysiology [49]. sICAM-1 belongs to the immunoglobulin superfamily, and it regulates the process of leukocytes adhesion to the endothelium as well as leukocyte migration [47]. The results of previous studies on soluble adhesion molecules in PE are conflicting. Some authors have revealed an increase in sP-selectin, sE-selectin, and sICAM-1 levels, while others have found no changes [50–53].

Up to date, there are a few data on the GH pathogenesis; most of the reports and studies were focused on preeclampsia itself, but since the clinical symptoms and the prognosis for mother and baby differ between GH and PE, it could be postulated that they are two different forms of hypertensive disorders related to pregnancy. So, the aim of this study was to evaluate the levels of adipokines such as adiponectin and leptin and the endothelium dysfunction markers: sICAM-1 and endogenous NOS inhibitor, ADMA, in pregnant women with gestational hypertension.

## 2. Study Design

### 2.1. Aim of the Study

The aim of this study was to evaluate the levels of adipokines such as adiponectin and leptin and the endothelium dysfunction markers: sICAM-1 and endogenous NOS inhibitor-ADMA, in pregnant women with gestational hypertension.

### 2.2. Characteristics of the Studied Groups

The random healthy patients who had prenatal visits in the outpatient clinic of the Department of Obstetrics and Perinatology of Medical University in Lublin were enrolled in the study. All of them gave informed consent to participate in the study, which had been previously approved by the Bioethical Review Board of the Medical University in Lublin. The blood samples from 168 women were taken between the 24^th^ and 34^th^ week of gestation; after having taken blood samples, all patients have been monitored toward the clinical and biochemical symptoms of pregnancy complications. Finally, 34 samples from GH patients and 32 samples from healthy pregnant women were analyzed; the remaining patients developed the pregnancy complications others than GH; thus, they were excluded from the analysis according to the study protocol. The inclusion criteria for the study were as follows: gestational age between the 24^th^ and 34^th^ week as determined on ultrasound before the 20^th^ week, first prenatal visit before the 8^th^ week, and singleton pregnancy. The exclusion criteria from the study were as follows: prepregnancy and gestational diabetes mellitus, chronic hypertension, preeclampsia, chronic renal disease, autoimmune diseases, intrauterine growth restriction, liver diseases, twin pregnancy, anticoagulant or corticosteroids therapy, inflammation, and infectious diseases. The patients with positive urine or vaginal culture were excluded from the study as well as cigarette smokers. The patients were finally included in the study group when gestational hypertension diagnosis according to ACOG criteria was made: the blood pressure value of ≥140/90 mmHg diagnosed after the 20^th^ week of gestation, in the absence of accompanying proteinuria or thrombocytopenia, renal and liver function impairment, with no other clinical symptoms (e.g., neurological, pulmonary) [1]. The assignment for the study and for the control group was performed after delivery when the final diagnosis of gestational hypertension was made in order to avoid the inclusion of patients with late-onset of preeclampsia.

### 2.3. Biochemical Methods

The blood samples for laboratory tests were taken fasting from all the patients between the 24^th^ and 34^th^ week of gestation together with samples for routinely performed laboratory tests. The samples were allowed to clot for at least 30 minutes before centrifugation at 1000*g*, which was continued for 30 minutes. Serum has been removed and then frozen at −70°C. All samples were stored up to 8 months, and this was similar for the study and the control group.

The leptin concentrations were measured by means of the sandwich enzyme immunoassay technique (Human Leptin Quantikine, R&D Systems Inc. Minneapolis, USA) as well as adiponectin (Human Adiponectin Quantikine, R&D Systems Inc., Minneapolis, USA), sICAM-1 (Human sICAM-1 Immunoassay, R&D Systems Inc., Minneapolis, USA), and ADMA (ADMA direct ELISA Kit, Immunodiagnostik AG, Bensheim, Germany). Body mass index (BMI) calculation was based on the weight measurement at the first prenatal visit before the 8^th^ week of gestation.

### 2.4. Statistical Methods

The study group was compared with the control one with respect to maternal age, gestational age at blood sampling, BMI, blood pressure, as well as leptin, adiponectin, sICAM-1, and ADMA concentrations. The correlations between the adipokines (leptin and adiponectin) and the endothelium dysfunction markers (sICAM-1 and ADMA) levels and BMI in the GH and the control group were analyzed.

The Statistica 5.5A for Windows (StatSoft, Poland) was used for data analysis. The elements of descriptive statistics were used. Data were presented as mean ± standard deviation. The Shapiro–Wilk test for normal distribution of data and one-tailed Student's *t*-test or (in unequal variance) the Cochran–Cox test (absence of normal distribution and nonparametric data) and the Mann–Whitney *U* test were performed. Spearman's rank test was employed for searching correlations between variables. The statistical significance was defined as *p* < 0.05.

## 3. Results and Discussion

### 3.1. Results

The patients with GH compared with healthy ones were characterized by significantly higher BMI (28.09 ± 7.90 vs. 22.34 ± 4.21 kg/m^2^, *p*=0.016), concentrations of leptin (45.89 ± 35.91 vs. 24.09 ± 24.40 ng/mL, *p*=0.006), and statistically insignificant higher levels of sICAM-1 (264.51 ± 50.99 vs. 232.56 ± 43.3 ng/ml, *p*=0.057). The average gestational age when the diagnosis of GH was made in the study group was 35.26 ± 3.48 weeks. There were no significant differences between groups in adiponectin (8.79 ± 8.67 vs. 7.90 ± 3.71 *μ*g/mL, *p*=0.46, NS) and ADMA (0.57 ± 0.26 vs. 0.60 ± 0.24 *μ*mol/L, *p*=0.68, NS) levels as well as the patients age and the gestational age at the blood samples collection (Table 1). The significant correlation between leptin levels and BMI value was observed only in the study group (*R* = 0.56, *p*=0.02) (Table 2).

## 4. Discussion

The aim of this study was to assess the levels of adipokines such as leptin and adiponectin and the endothelium dysfunction markers: sICAM-1 and endogenous NOS inhibitor, ADMA, in pregnant women with gestational hypertension. The authors of the present research acknowledged the 24^th^ week as the lowest limit of gestation for the patient inclusion as it allowed to exclude women with an uncertain diagnosis (for example, chronic hypertension diagnosed for the first time in pregnancy). Since severe hypertension after the 34^th^ week, mainly in the case of preeclampsia, could be the indication for delivery, the gestational age of the 34^th^ week was adopted as the upper limit for the inclusion. There are few studies which focus only on GH as a separate form of hypertension in pregnancy; the vast majority of reports present the data on adipokines in PE; thus, the difference between studied groups (PE, only GH or PE and GH together) may be important while discussing the results.

We have found that pregnant patients with GE were characterized by significantly higher concentrations of leptin and with higher BMI compared with healthy ones. Our observations are similar to the results of previous studies conducted in preeclamptic patients which have suggested a positive correlation between elevated serum leptin levels and preeclampsia [26, 27, 54, 55].

The opposite conclusions have been presented by Eleuterio et al. They studied patients with PE, GH, and healthy ones, and they have found no differences in leptin concentrations between groups. The groups were similar in respect of BMI, but only patients with BMI < 30 kg/m^2^ were included into the study, what could explain the discrepancies between the aforementioned report and the results of our research where BMI was neither the criterion of inclusion nor exclusion [56]. These differences could also be the result of different gestational ages at the blood sampling.

Taylor et al. measured the leptin concentration in early pregnancy (9–16th week), and they have found that it was significantly higher in women with subsequent PE development. In their study, women with PE were characterized by overweight and obesity, so the authors have concluded that leptin could be a possible mediator of the association between overweight and obesity and preeclampsia [57]. This observation has been confirmed by the results of our research. It has been shown that serum leptin levels are increased in obesity, and higher BMI has been found to be associated with PE and, according to the results of our study, also with gestational hypertension [58, 59].

Chrelias et al. have found that overweight and obesity were connected with PE development and higher levels of leptin. They are of the opinion that increased serum leptin concentrations reflect rather maternal obesity and overweight than are the independent risk factor for PE development [60]. In obese pregnant women, an increase in placental leptin resistance has been reported due to syncytiotrophoblast downregulation of leptin receptors during states of maternal hyperleptinemia [61].

The study conducted on an animal model has revealed that chronic hyperleptinemia increases blood pressure and, in the absence of additional metabolic conditions, it could be an important link between obesity and the development of hypertension during pregnancy [62]. Additionally, it has been found that hyperleptinemia was present also in nonobese PE patients what is suggestive that the placenta is another, except adipose tissue, an important source of leptin [63]. Indeed, the placental leptin gene expression and proteins are elevated in PE. However, it is unknown to what extent leptin derived from adipose and/or placental tissues is involved in pregnancy-related hypertension [64].

The postulated mechanisms for leptin-induced hypertension development involve stimulating phosphorylation and activation of mitogen-activated protein kinases and phosphatidylinositol-3 kinase to enhance the proliferation and migration of vascular smooth muscle cells, intensifying the effects of angiotensin II through modulating the sympathetic nervous system, initiating leukocyte and macrophage recruitment to the endothelial wall and inducing the synthesis of reactive oxygen species in endothelial cells [65].

According to the published reports, hyperleptinemia appears to develop in preeclamptic women during the first and second trimester [66, 67].

Our study has revealed that higher leptin levels in maternal serum proceed the GH development: the blood samples were collected in the 24^th^–34^th^ week of pregnancy while the average gestational age when GH was diagnosed was 35.26 ± 3.48 weeks.

According to the results of the present study, it may be postulated that hyperleptinemia associated with obesity may be involved in gestational hypertension development.

Most reports have shown the significantly higher adiponectin levels in PE patients [53, 55, 65], but there are also some publications what have presented the opposite results [36, 68].

We have found no difference in adiponectin concentrations between patients with GH and healthy ones, although patients with GH had significantly higher BMI. No correlations between adiponectin and BMI have been observed both in the study and the control group.

The similar observations have been presented by Song et al. who have found no differences in adiponectin levels between PE and healthy patients; PE women had significantly higher BMI [69].

Our results are opposite to those presented by Eleutario et al. who have shown higher adiponectin levels in patients with PE compared with healthy ones, but they included in their study only patients with BMI < 30 kg/m^2^ [70]. We did not use BMI as a criterion of the inclusion or exclusion from the study; the average BMI of the patients with GH enrolled in our research was 28.09 kg/m^2^ which is suggestive that also obese patients (>30 kg/m^2^) were assessed. The significant increase in adiponectin levels in patients with PE might be considered as a protective mechanism, improving vascular function and insulin sensitivity [28, 34, 35, 58, 71]. It is speculated that adiponectin by suppression of inflammation processes and enhancing NO synthesis within the vascular wall could preserve the normal blood pressure [32, 33]. The elevated adiponectin levels in PE could also be explained with the adiponectin resistance what has been observed in animal models [72].

Additionally, some authors have suggested that the elevated adiponectin levels in the first and second trimester might predict PE development [73], whereas others have shown opposite results: GH and PE development could be proceeded by lower concentrations of adiponectin [12]. There are also reports showing the lack of relationship between adiponectin levels and subsequent PE development [74].

No correlations between adiponectin and BMI have been observed either in GH or in the control group in the present study. The published data on this relationship is inconclusive. Song et al. have found that adiponectin levels were unrelated to BMI in PE pregnant women [69], as well as Eleuterio et al. who have revealed that adiponectin levels were correlated with BMI only in healthy pregnant women, but not in PE women [56]. According to the results published by Hendler et al., it seems that BMI is crucial: they have revealed that BMI ≥ 25 kg/m^2^ was connected with the decrease in adiponectin levels, whereas the increase in concentrations of this adipokine characterized patients with PE of normal weight [58]. The comparable adiponectin level in GH and healthy patients and the lack of its correlation with BMI presented in this study may indicate the occurrence of a protective mechanism in pregnancy, maintaining its concentration and preserving from the consequences of its decrease observed in nonpregnant, overweight, and obese patients.

There are a lot of reports in the literature so far on sVCAM-1 as the marker of the endothelial dysfunction, and we focused only on ADMA and sICAM-1 level because the data on these markers in GH are not only limited but also conflicting.

sICAM-1 is released during inflammation, and it is involved in both adhesions of leukocytes to the endothelium and leukocyte migration; thus, its increased plasma levels seem to reflect the impaired endothelium function [75].

We have found higher concentrations of this molecule, although not statistically significant, in the study group compared with healthy pregnant women what could be suggestive that some mild impairment of the endothelium function might occur in GH. The lack of correlation of sICAM-1 with BMI in GH and healthy patients suggests that in this hypertensive pregnancy complication, overweight or obesity has no significant impact on the endothelial function.

Previous studies on soluble adhesion molecules in PE have yielded conflicting results what might be explained with the PE complexity [76]. If the hypothesis of different nature of GH and PE is considered, contradictory results could be expected. Fei et al. have reported the similar results to ours: they have found higher sICAM-1 concentrations in pregnancy-induced hypertension (PIH) than in healthy patients, but they were lower compared with levels in the PE group [77]. Zhao et al. assessing sICAM-1 levels in the group of PE, GH, and healthy patients have revealed that the concentrations of this molecule were highest in the PE group and higher in the GH group than in the control one. The PE patients had also highest BMI (higher compared to GH patients). The blood samples were taken in the second trimester which is very similar to the protocol of our study. The authors have concluded that PE and GH are associated with inflammation processes within the vascular wall what was confirmed by increased sICAM-1 levels in these patients [78].

According to the results of the study cited above, the deeper endothelial dysfunction could be expected in PE than in GH, and it might explain partially a more serious clinical course of PE with the impairment of internal organs. The opposite observations have been presented by Rios et al. and Valencia-Ortega et al. who have found no significant increase in sICAM-1 levels in early and late-onset PE and severe PE [79, 80].

We aimed to assess the levels of ADMA, the inhibitor of NOS, in patients with gestational hypertension. ADMA is a competitive inhibitor of L-arginine for all NOS isoforms. Intracellular ADMA inhibits NOS activity and limits the cellular uptake of L-arginine, which eventually results in reduced NO synthesis [81]. Nitric oxide, as the main vasoactive agent in the placenta, impacts on trophoblast invasion [44]. If impaired trophoblast invasion is considered as one of the possible reasons for PE development, the limited NO synthesis caused by elevated ADMA concentrations seems to be involved in PE pathogenesis. No statistically significant differences have been found between GH patients and the healthy ones in the present study; thus, it could be speculated that ADMA is not involved in GH development, suggesting that NO synthesis is not disturbed in this complication of pregnancy. This observation might support the hypothesis of the possible different nature of PE and GH. The majority of previously published reports have presented the opposite results, but studies were conducted in patients with preeclampsia what, taken into consideration that PE and GH could be two different conditions, might justify these discrepancies.

The available data have supported the thesis that ADMA levels in PE are increased although Noorbakhsh et al. have found no significantly higher ADMA concentrations in PE patients [82]. However, the authors of the recently published meta-analysis have concluded that the concentrations of ADMA, the remarkable marker of the endothelial dysfunction, are significantly higher in PE, mainly early-onset PE, than in healthy pregnant patients. They are of the opinion that ADMA may play a major role in the PE development [83].

ADMA levels have been shown to increase even before the development of preeclampsia, suggesting that increased ADMA may be linked with the occurrence of preeclampsia in high-risk women [84]. We have observed no correlation between ADMA levels and BMI either in the GH or control group although the previous research has revealed such a connection in preeclamptic patients [83]. This could be the additional argument supporting the hypothesis that GH and PE are of different pathophysiological origin.

The limitations of the present study are connected with the relatively limited size of the studied groups. As this research was focused on gestational hypertension only and the available data are conflicting, the interpretation of the results is demanding. Since it seems that gestational hypertension is separate from preeclampsia form of hypertensive diseases induced by pregnancy, the need for further investigations on GH pathogenesis and the role of adipokines and the endothelium dysfunction markers is warranted.

## 5. Conclusions

The higher levels of leptin in pregnant women with gestational hypertension may be suggestive of the role of leptin in GH development. As the patients in the GH group had higher BMI, hyperleptinemia may link obesity with gestational hypertension. The significance of leptin as the predictive marker of GH development could be implied. It could be postulated that the higher levels of sICAM-1 in the GH patients, although not statistically significant, could reflect some impairment of the endothelium function occurring in GH regardless of BMI. The comparable adiponectin levels in GH and healthy pregnant patients and the lack of its correlation with BMI may indicate the occurrence of a protective mechanism in pregnancy, maintaining its concentration and preserving from the consequences of the decrease in its levels in overweight and obese patients. As ADMA levels were similar in GH and healthy pregnant women, ADMA seems not to be involved in GH pathogenesis, suggesting that NO synthesis is not impaired in this pregnancy complication.

Since the data on the gestational hypertension pathogenesis and their correlations with adipokines and markers of the endothelium dysfunction are limited, further studies on this issue are warranted.

## Figures and Tables

**Table 1:** The adipokines and the endothelium dysfunction markers in the gestational hypertension group and in the control group.

	GH group, *n* = 34	Control group, *n* = 32	*p*
Age	28.35 ± 4.75	28.50 ± 4.95	NS
Gestational age (hbd)	30.88 ± 0.71	29.13 ± 2.96	NS
BMI (kg/m^2^)	28.09 ± 7.90	22.34 ± 4.21	*p*=0.016
Blood pressure			
Systolic (mmHg)	159.17 ± 12.16	123.83 ± 13.97	*p* < 0.0001
Diastolic (mmHg)	101.18 ± 9.75	73.00 ± 10.51	*p* < 0.0001
Leptin (ng/mL)	45.89 ± 35.91	24.09 ± 24.40	*p*=0.006
Adiponectin (*μ*g/mL)	8.79 ± 8.67	7.90 ± 3.71	NS
sICAM-1 (ng/mL)	264.51 ± 50.99	232.56 ± 43.30	*p*=0.057
ADMA (*μ*mol/L)	0.57 ± 0.26	0.60 ± 0.24	NS

GH: gestational hypertension; BMI: body mass index; sICAM-1: soluble intercellular adhesion molecule-1; ADMA: asymmetric dimethylarginine; *p*: statistical significance; NS: statistically not significant. Statistical data analysis: the Shapiro–Wilk test, one-tailed Student's *t*-test, Cochran–Cox test, and the Mann–Whitney *U* test.

**Table 2:** The correlations between the adipokines and the endothelium dysfunction marker levels and BMI in the GH group and the control group.

	GH group					Control group				
	BMI	Leptin	Adiponectin	sICAM-1	ADMA	BMI	Leptin	Adiponectin	sICAM-1	ADMA
Leptin	*R* = 0.56, *p*=0.02	—	*R* = 0.18, NS	*R* = 0.06, NS	*R* = 0.06, NS	*R* = −0.03, NS	—	*R* = 0.19, NS	*R* = 0.08, NS	*R* = 0.08, NS
Adiponectin	*R* = −0.18, NS	*R* = 0.18 NS	—	*R* = 0.01, NS	*R* = −0.03, NS	*R* = 0.23, NS	*R* = 0.19, NS	—	*R* = 0.15, NS	*R* = 0.48, NS
sICAM-1	*R* = 0.40, NS	*R* = 0.06, NS	*R* = 0.01, NS	—	*R* = 0.18, NS	*R* = −0.40, NS	*R* = 0.08, NS	*R* = 0.15, NS	—	*R* = 0.12, NS
ADMA	*R* = 0.38, NS	*R* = 0.06, NS	*R* = −0.03, NS	*R* = 0.18, NS	—	*R* = 0.16, NS	*R* = 0.08, NS	*R* = 0.48, NS	*R* = 0.12, NS	—

GH: gestational hypertension; BMI: body mass index; sICAM-1: soluble intercellular adhesion molecule-1; ADMA: asymmetric dimethylarginine; *R*: Spearman correlation's coefficient; *p*: statistical significance; NS: statistically not significant. Statistical data analysis: Spearman's rank test.

## Data Availability

The statistical data used to support the findings of this study are included in the article.

## References

[1] American College of Obstetricians and Gynecologists (2013). *Hypertension in Pregnancy*.

[2] Mosca L., Benjamin E. J., Berra K. (2011). Effectiveness-based guidelines for the prevention of cardiovascular disease in women—2011 update. *Journal of the American College of Cardiology*.

[3] Khan K. S., Wojdyla D., Say L., Gülmezoglu A. M., Van Look P. F. (2006). WHO analysis of causes of maternal death: a systematic review. *The Lancet*.

[4] Davis E. F., Lazdam M., Lewandowski A. J. (2012). Cardiovascular risk factors in children and young adults born to preeclamptic pregnancies: a systematic review. *Pediatrics*.

[5] McDonald S. D., Malinowski A., Zhou Q., Yusuf S., Devereaux P. J. (2008). Cardiovascular sequelae of preeclampsia/eclampsia: a systematic review and meta-analyses. *American Heart Journal*.

[6] Duckitt K., Harrington D. (2005). Risk factors for pre-eclampsia at antenatal booking: systematic review of controlled studies. *BMJ*.

[7] Mbah A., Kornosky J., Kristensen S. (2010). Super-obesity and risk for early and late pre-eclampsia. *BJOG: An International Journal of Obstetrics & Gynaecology*.

[8] Sankaralingam S., Arenas I. A., Lalu M. M., Davidge S. T. (2006). Preeclampsia: current understanding of the molecular basis of vascular dysfunction. *Expert Reviews in Molecular Medicine*.

[9] Redman C. W. G., Sargent I. L. (2010). Immunology of pre-eclampsia. *American Journal of Reproductive Immunology*.

[10] Quyyumi A. A. (1998). Endothelial function in health and disease: new insights into the genesis of cardiovascular disease. *The American Journal of Medicine*.

[11] Lu D., Yang X., Wu Y., Wang H., Huang H., Dong M. (2006). Serum adiponectin, leptin and soluble leptin receptor in pre-eclampsia. *International Journal of Gynecology & Obstetrics*.

[12] D’Anna R., Baviera G., Corrado F., Giordano D., Di Benedetto A., Jasonni V. M. (2005). Plasma adiponectin concentration in early pregnancy and subsequent risk of hypertensive disorders. *Obstetrics & Gynecology*.

[13] Agarwal A., Aponte-Mellado A., Premkumar B. J., Shaman A., Gupta S. (2012). The effects of oxidative stress on female reproduction: a review. *Reproductive Biology and Endocrinology*.

[14] Antuna-Puente B., Feve B., Fellahi S., Bastard J.-P. (2008). Adipokines: the missing link between insulin resistance and obesity. *Diabetes & Metabolism*.

[15] Lappas M., Yee K., Permezel M., Rice G. E. (2005). Release and regulation of leptin, resistin and adiponectin from human placenta, fetal membranes, and maternal adipose tissue and skeletal muscle from normal and gestational diabetes mellitus-complicated pregnancies. *Journal of Endocrinology*.

[16] Ahima R. S., Flier J. S. (2000). Leptin. *Annual Review of Physiology*.

[17] Castellucci M., De Matteis R., Meisser A. (2000). Leptin modulates extracellular matrix molecules and metalloproteinases: possible implications for trophoblast invasion. *Molecular Human Reproduction*.

[18] Otero M., Lago R., Lago F. (2005). Leptin, from fat to inflammation: old questions and new insights. *FEBS Letters*.

[19] Henson M. C., Castracane V. D. (2006). Leptin in pregnancy: an update1. *Biology of Reproduction*.

[20] Balland E., Cowley M. A. (2015). New insights in leptin resistance mechanisms in mice. *Frontiers in Neuroendocrinology*.

[21] Poniedziałek-Czajkowska E., Mierzyński R., Słodzińska M., Dłuski D., Leszczyńska-Gorzelak B. (2018). Adipokines ad C-peptide in overweight and obese pregnant women. *Ginekologia Polska*.

[22] Hiraoka J., Hosoda K., Ogawa Y. (1997). Augmentation ofobese(ob) gene expression and leptin secretion in obese spontaneously hypertensive rats (obese SHR or koletsky rats). *Biochemical and Biophysical Research Communications*.

[23] Ibrahim H. S., Omar E., Froemming G. R. A., Singh H. J. (2013). Leptin increases blood pressure and markers of endothelial activation during pregnancy in rats. *BioMed Research International*.

[24] Huby A.-C., Otvos L., Belin de Chantemèle E. J. (2016). Leptin induces hypertension and endothelial dysfunction via aldosterone-dependent mechanisms in obese female mice. *Hypertension*.

[25] Matarese G., Moschos S., Mantzoros C. S. (2005). Leptin in immunology. *The Journal of Immunology*.

[26] Laivuori H., Gallaher M. J., Collura L. (2006). Relationships between maternal plasma leptin, placental leptin mRNA and protein in normal pregnancy, pre-eclampsia and intrauterine growth restriction without pre-eclampsia. *MHR: Basic Science of Reproductive Medicine*.

[27] Mise H., Sagawa N., Matsumoto T. (1998). Augmented placental production of leptin in preeclampsia: possible involvement of placental hypoxia. *Journal of Clinical Endocrinology & Metabolism*.

[28] Miehle K., Stepan H., Fasshauer M. (2012). Leptin, adiponectin and other adipokines in gestational diabetes mellitus and pre-eclampsia. *Clinical Endocrinology*.

[29] Retnakaran A., Retnakaran R. (2012). Adiponectin in pregnancy: implications for health and disease. *Current Medicinal Chemistry*.

[30] Goldstein B. J., Scalia R. G., Ma X. L. (2009). Protective vascular and myocardial effects of adiponectin. *Nature Clinical Practice Cardiovascular Medicine*.

[31] Ouchi N., Kihara S., Funahashi T. (2003). Reciprocal association of C-reactive protein with adiponectin in blood stream and adipose tissue. *Circulation*.

[32] Rasouli N., Kern P. A. (2008). Adipocytokines and the metabolic complications of obesity. *The Journal of Clinical Endocrinology & Metabolism*.

[33] Catalano P. M., Hoegh M., Minium J. (2006). Adiponectin in human pregnancy: implications for regulation of glucose and lipid metabolism. *Diabetologia*.

[34] Kajantie E., Kaaja R., Ylikorkala O., Andersson S., Laivouri H. (2005). Adiponectin concentrations in maternal serum: elevated in preeclampsis but unrelated to insulin sensitivity. *Journal of the Society for Gynecologic Investigation*.

[35] Naruse K., Yamasaki M., Umekage H., Sado T., Sakamoto Y., Morikawa H. (2005). Peripheral blood concentrations of adiponectin, an adipocyte-specific plasma protein, in normal pregnancy and preeclampsia. *Journal of Reproductive Immunology*.

[36] D’Anna R., Baviera G., Corrado F. (2006). Adiponectin and insulin resistance in early- and late-onset pre-eclampsia. *BJOG: An International Journal of Obstetrics and Gynaecology*.

[37] Cortelazzi D., Corbetta S., Ronzoni S. (2007). Maternal and foetal resistin and adiponectin concentrations in normal and complicated pregnancies. *Clinical Endocrinology*.

[38] Roberts J. (1998). Endothelial dysfunction in preeclampsia. *Seminars in Reproductive Medicine*.

[39] Ali S. M., Khalil R. A. (2015). Genetic, immune and vasoactive factors in the vascular dysfunction associated with hypertension in pregnancy. *Expert Opinion on Therapeutic Targets*.

[40] Shah D. A., Khalil R. A. (2015). Bioactive factors in uteroplacental and systemic circulation link placental ischemia to generalized vascular dysfunction in hypertensive pregnancy and preeclampsia. *Biochemical Pharmacology*.

[41] Ali Z., Khaliq S., Zaki S., Ahmad H., Lone K. (2019). Altered expression of vascular endothelial growth factor, vascular endothelial growth factor receptor-1, vascular endothelial growth factor receptor-2, and soluble Fms-like tyrosine kinase-1 in peripheral blood mononuclear cells from normal and preeclamptic pregnancies. *Chinese Journal of Physiology*.

[42] Cai H., Harrison D. G. (2000). Endothelial dysfunction in cardiovascular diseases: the role of oxidant stress. *Circulation Research*.

[43] Ahmad A., Dempsey S., Daneva Z. (2018). Role of nitric oxide in the cardiovascular and renal systems. *International Journal of Molecular Sciences*.

[44] Krause B. J., Hanson M. A., Casanello P. (2011). Role of nitric oxide in placental vascular development and function. *Placenta*.

[45] Kielstein J. T., Impraim B., Simmel S. (2004). Cardiovascular effects of systemic nitric oxide synthase inhibition with asymmetrical dimethylarginine in humans. *Circulation*.

[46] Sibal L., Agarwal S. C., Home P. D., Boger R. H. (2010). The role of asymmetric dimethylarginine (ADMA) in endothelial dysfunction and cardiovascular disease. *Current Cardiology Reviews*.

[47] López-Alarcón M., Montalvo-Velarde I., Vital-Reyes V., Hinojosa-Cruz J., Leaños-Miranda A., Martínez-Basila A. (2015). Serial determinations of asymmetric dimethylarginine and homocysteine during pregnancy to predict pre-eclampsia: a longitudinal study. *BJOG: An International Journal of Obstetrics & Gynaecology*.

[48] Alpoim P. N., Godoi L. C., Freitas L. G., Gomes K. B., Dusse L. M. (2013). Assessment of L-arginine asymmetric 1 dimethyl (ADMA) in early-onset and late-onset (severe) preeclampsia. *Nitric Oxide*.

[49] Farzadnia M., Ayatollahi H., Hasan-Zade M., Rahimi H. R. (2013). A comparative study of serum level of vascular cell adhesion molecule-1 (sVCAM-1), intercellular adhesion molecule-1(ICAM-1) and high sensitive C—reactive protein (hs-CRP) in normal and pre-eclamptic pregnancies. *Iranian Journal of Basic Medical Sciences*.

[50] Kim S.-Y., Ryu H.-M., Yang J. H. (2004). Maternal serum levels of VCAM-1, ICAM-1 and E-selectin in preeclampsia. *Journal of Korean Medical Science*.

[51] Higgins J. R., Papayianni A., Brady H. R., Darling M. R. N., Walshe J. J. (1998). Circulating vascular cell adhesion molecule-1 in pre-eclampsia, gestational hypertension, and normal pregnancy: evidence of selective dysregulation of vascular cell adhesion molecule-1 homeostasis in pre-eclampsia. *American Journal of Obstetrics and Gynecology*.

[52] Krauss T., Osmers R., Beran J., Diedrich F., Fleckenstein G., Kuhn W. (1998). Soluble adhesion molecules in patients with pre-eclampsia. *Zentralblatt Für Gynäkologie*.

[53] Daniel Y., Kupferminc M. J., Baram A. (1998). Plasma soluble endothelial selectin is elevated in women with pre-eclampsia. *Human Reproduction*.

[54] Ouyang Y., Chen H., Chen H. (2007). Reduced plasma adiponectin and elevated leptin in pre-eclampsia. *International Journal of Gynecology & Obstetrics*.

[55] Molvarec A., Szarka A., Walentin S. (2011). Serum leptin levels in relation to circulating cytokines, chemokines, adhesion molecules and angiogenic factors in normal pregnancy and preeclampsia. *Reproductive Biology and Endocrinology*.

[56] Eleuterio N. M., Palei A. C. T., Rangel Machado J. S., Tanus-Santos J. E., Cavalli R. C., Sandrim V. C. (2014). Correlations between circulating levels of adipokines and anti-angiogenic factors in women with BMI <30 and a late-onset preeclampsia. *Hypertension in Pregnancy*.

[57] Taylor B. D., Ness R. B., Olsen J. (2015). Serum leptin measured in early pregnancy is higher in women with preeclampsia compared with normotensive pregnant women. *Hypertension*.

[58] Hendler I., Blackwell S. C., Mehta S. H. (2005). The levels of leptin, adiponectin, and resistin in normal weight, overweight, and obese pregnant women with and without preeclampsia. *American Journal of Obstetrics and Gynecology*.

[59] Bodnar L. M., Catov J. M., Klebanoff M. A., Ness R. B., Roberts J. M. (2007). Prepregnancy body mass index and the occurrence of severe hypertensive disorders of pregnancy. *Epidemiology*.

[60] Chrelias G., Makris G.-M., Papanota A.-M. (2016). Serum inhibin and leptin: risk factors for pre-eclampsia?. *Clinica Chimica Acta*.

[61] Farley D. M., Choi J., Dudley D. J. (2010). Placental amino acid transport and placental leptin resistance in pregnancies complicated by maternal obesity. *Placenta*.

[62] Palei A. C., Spradley F. T., Granger J. P. (2015). Chronic hyperleptinemia results in the development of hypertension in pregnant rats. *American Journal of Physiology-Regulatory, Integrative and Comparative Physiology*.

[63] Masuzaki H., Ogawa Y., Sagawa N. (1997). Nonadipose tissue production of leptin: leptin as a novel placenta-derived hormone in humans. *Nature Medicine*.

[64] Haugen F., Ranheim T., Harsem N. K., Lips E., Staff A. C., Drevon C. A. (2006). Increased plasma levels of adipokines in preeclampsia: relationship to placenta and adipose tissue gene expression. *American Journal of Physiology-Endocrinology and Metabolism*.

[65] Tsai J.-P. (2017). The association of serum leptin levels with metabolic diseases. *Tzu Chi Medical Journal*.

[66] Samolis S., Papastefanou I., Panagopoulos P., Galazios G., Kouskoukis A., Maroulis G. (2010). Relation between first trimester maternal serum leptin levels and body mass index in normotensive and pre-eclamptic pregnancies—role of leptin as a marker of pre-eclampsia: a prospective case-control study. *Gynecological Endocrinology*.

[67] Papastefanou I., Samolis S., Panagopoulos P. (2010). Correlation between maternal first trimester plasma leptin levels and birth weight among normotensive and preeclamptic women. *The Journal of Maternal-Fetal & Neonatal Medicine*.

[68] O’Sullivan A. J., Kriketos A. D., Martin A., Brown M. A. (2006). Serum adiponectin levels in normal and hypertensive pregnancy. *Hypertension in Pregnancy*.

[69] Song Y., Gao J., Qu Y., Wang S., Wang X., Liu J. (2016). Serum levels of leptin, adiponectin and resistin in relation to clinical characteristics in normal pregnancy and preeclampsia. *Clinica Chimica Acta*.

[70] Eleuterio N. M., Palei A. C. T., Rangel Machado J. S., Tanus-Santos J. E., Cavalli R. C., Sandrim V. C. (2015). Positive correlations between circulating adiponectin and MMP2 in preeclampsia pregnant. *Pregnancy Hypertension: An International Journal of Women’s Cardiovascular Health*.

[71] Ramsay J. E., Jamieson N., Greer I. A., Sattar N. (2003). Paradoxical elevation in adiponectin concentrations in women with preeclampsia. *Hypertension*.

[72] Lin H. V., Kim J.-Y., Pocai A. (2007). Adiponectin resistance exacerbates insulin resistance in insulin receptor transgenic/knockout mice. *Diabetes*.

[73] Nanda S., Yu C. K. H., Giurcaneanu L., Akolekar R., Nicolaides K. H. (2011). Maternal serum adiponectin at 11–13 weeks of gestation in preeclampsia. *Fetal Diagnosis and Therapy*.

[74] Odden N., Mørkrid L. (2007). High molecular weight adiponectin dominates in cord blood of newborns but is unaffected by pre-eclamptic pregnancies. *Clinical Endocrinology*.

[75] Freeman D. J., McManus F., Brown E. A. (2004). Short- and long-term changes in plasma inflammatory markers associated with preeclampsia. *Hypertension*.

[76] Dusse L. M., Rios D. R. A., Pinheiro M. B., Cooper A. J., Lwaleed B. A. (2011). Pre-eclampsia: relationship between coagulation, fibrinolysis and inflammation. *Clinica Chimica Acta*.

[77] Fei X., Hongxiang Z., Qi C., Daozhen C. (2012). Maternal plasma levels of endothelial dysfunction mediators including AM, CGRP, sICAM-1 and tHcy in pre-eclampsia. *Advances in Clinical and Experimental Medicine*.

[78] Zhao J., Zheng D.-Y., Yang J.-M. (2016). Maternal serum uric acid concentration is associated with the expression of tumour necrosis factor-*α* and intercellular adhesion molecule-1 in patients with preeclampsia. *Journal of Human Hypertension*.

[79] Rios D. R. A., Alpoim P. N., Godoi L. C. (2016). Increased levels of sENG and sVCAM-1 and decreased levels of VEGF in severe preeclampsia. *American Journal of Hypertension*.

[80] Valencia-Ortega J., Zárate A., Saucedo R., Hernández-Valencia M., Cruz J. G., Puello E. (2018). Placental proinflammatory state and maternal endothelial dysfunction in preeclampsia. *Gynecologic and Obstetric Investigation*.

[81] Huang L.-T., Hsieh C.-S., Chang K.-A., Tain Y.-L. (2012). Roles of nitric oxide and asymmetric dimethylarginine in pregnancy and fetal programming. *International Journal of Molecular Sciences*.

[82] Noorbakhsh M., Kianpour M., Nematbakhsh M. (2013). Serum levels of asymmetric dimethylarginine, vascular endothelial growth factor, and nitric oxide metabolite levels in preeclampsia patients. *ISRN Obstetrics and Gynecology*.

[83] Németh B., Murányi E., Hegyi P. (2018). Asymmetric dimethylarginine levels in preeclampsia—systematic review and meta-analysis. *Placenta*.

[84] Savvidou M. D., Hingorani A. D., Tsikas D., Frölich J. C., Vallance P., Nicolaides K. H. (2003). Endothelial dysfunction and raised plasma concentrations of asymmetric dimethylarginine in pregnant women who subsequently develop pre-eclampsia. *The Lancet*.

